# Policy for Prevention of a Retained Sponge after Vaginal Delivery

**DOI:** 10.1155/2012/317856

**Published:** 2012-01-24

**Authors:** David J. Garry, Sandra Asanjarani, Donna M. Geiss

**Affiliations:** ^1^Department of Obstetrics, Gynecology and Women's Health, Albert Einstein College of Medicine at Yeshiva University, Jacobi Medical Center, 1400 Pelham Parkway South, Bronx, NY 10461, USA; ^2^Department of Obstetrics, Gynecology and Women's Health, Montefiore Medical Center, North Division, 600 East 233rd Street, Bronx, NY 10466, USA

## Abstract

*Background*. Policies for sponge count are not routine practice in most labor and delivery rooms. Ignored or hidden retained vaginal foreign bodies has potentially significant health care morbidity. *Case*. This was a case of a retained vaginal sponge following an uncomplicated spontaneous vaginal delivery. Delivery room policy resulted in the discovery of the sponge on X-ray when an incorrect sponge count occurred and physical exam did not find the sponge. *Conclusion*. This emphasizes the use of protocols to enhance patient safety and prevent medical error.

## 1. Introduction

Error in medicine has been commonly reported and can lead to harm [1]. Retention of surgical instruments or sponges has been considered an avoidable medical error [2]. Due to possible asymptomatic patients, the incidence of retained sponges can vary from 1 in 100 to 1 in 5000 procedures [2, 3]. Open abdominal procedures in obstetrics, cesarean delivery the most frequent, occur in the operating room and a formal 2 person sponge count is routine. Vaginal deliveries typically occur in specialized labor and delivery rooms and formal sponge counts following delivery are uncommonly performed. This case illustrates that formal sponge counts following vaginal delivery can effectively prevent a retained foreign body.

## 2. Case

The patient was a 15-year-old nulliparous female with an unsure last menstrual period and an estimated delivery date of March 15, 2010. Her past medical, surgical, and social history was unremarkable and she lived with her mother and two siblings. Prenatal care was initiated 28 weeks and complicated by Chlamydia cervicitis which was treated with azithromycin.

She presented to labor and delivery at 39 1/7 weeks for evaluation and noted to have oligohydramnios on ultrasound exam. She was admitted and underwent induction of labor using a cervical Foley catheter. After the Foley catheter fell out, labor augmentation with oxytocin was required and epidural anesthesia was provided for pain management. She progressed in labor and delivered vaginally, 23 hours after initiation of labor induction. The baby weighed 2435 g with Apgars of 9 at one minute and 9 at 5 minutes and normal umbilical cord gases. The placenta spontaneously delivered, and evaluation of the perineum demonstrated a small periurethral laceration and 2 deep anterior vaginal lacerations. The lacerations were uneventfully repaired with absorbable suture.

**Figure 1:**
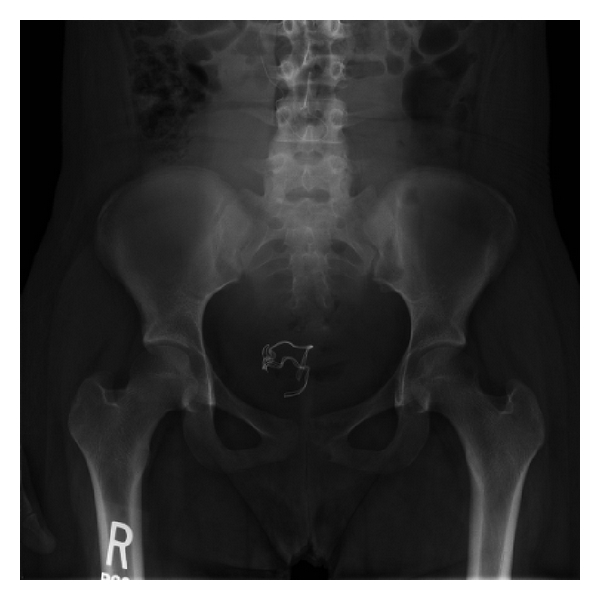
Postdelivery X-ray of the pelvis demonstrating two curvilinear triangular-shaped structures composed of 2 radiopaque threads in the vaginal/rectal area.

Following protocol for all deliveries in our institution, a sponge count was done immediately after delivery (Table 1). The sponge count was not correct and following protocol, a physical exam was performed by the obstetrician. The obstetrician could not locate the missing sponge and the delivery room was searched without resolve. A pelvic X-ray was obtained demonstrating two curvilinear triangular-shaped structures composed of radiopaque threads in the vaginal/rectal area. There was a wire overlying the lumbar spine which was consistent with an epidural catheter (Figure 1). 

Repeated exam of the patient retrieved a sponge deep in the posterior fornix. The remainder of the postpartum period was uneventful. The patient opted for oral contraception and received information on condom use, sexually transmitted diseases, and on the importance of keeping her postpartum visit.

## 3. Discussion

The exact incidence of retained foreign bodies following vaginal delivery remains unknown. In a recent retrospective-case review, 11 cases of sponge retention following episiotomy or vaginal lacerations repair were reported during the study period [4]. Many of these were discovered on patient self-examination. Vaginal delivery is a bloody procedure and the potential for a retained sponge, as in our case, is high despite a physical exam being performed. The odds of a retained foreign body are increased 100 times especially with a discrepancy in counts [5]. Our policy of counting sponges at the time of delivery and the requirement for imaging if there is any discrepancy allowed for identification of the retained sponge in this case.

 The primary method for assessment of retained foreign bodies is radiographic evaluation when there are differences in instrument or sponge counts. Alternative methods, not involving X-ray radiation, have been evaluated. The use of automated counting using bar-coded surgical sponges has shown improvement in detection of miscounted and misplaced sponges and was well tolerated by surgical staff members [6]. The United States Food and Drug Administration has currently approved 3 technologies for the identification of sponges, which include a bar-coded sponge detection system, a radiofrequency-tagged sponge detection system, and passive radiofrequency identification tagged sponge detection system; they have all demonstrated potential cost saving benefits [7].

 Anecdotal stories of performing an exam and turning the glove inside out, thus hiding the retained sponge, have been described whenever a vaginal sponge is found and discussed. The practice of counting sponges during obstetrical procedures should be routine and may help reduce postpartum morbidity [4]. This practice has been incorporated at our facility utilizing sponges with radiopaque threads in the delivery packs and allowing for physical exam of the patient and immediate environment before exposure to X-ray occurs.

## Figures and Tables

**Table 1:** Institutional policy for vaginal delivery sponge count.

(1) The count as follows:	
(a) before the procedure,	
(b) after the completion of the procedure.	
(2) The delivery room nurse will record the counts on the Vaginal Delivery Bundle Count Record and document the accuracy of the count in the nurse's notes section of the EMR.	
(3) The delivery room team is notified immediately if the count is found to be incorrect.	
(4) If the count is noted to be incorrect,	
(a) repeat the count;	
(b) the vaginal vault should be thoroughly examined by the physician or midwife;	
(c) The delivery room should be searched for the missing item, including trash containers, linen hampers, and waste buckets, under table and around the room;	
(d) If the missing item remains unaccounted for, an X-ray of the pelvis is to be performed;	
(e) The count is to be documented as incorrect;	
(f) An Occurrence Report should be completed and the administrative supervisor will be informed.	
